# The Evolving Microbial Paradigm in Acne

**DOI:** 10.3390/biom16030430

**Published:** 2026-03-13

**Authors:** Maurice A. M. van Steensel

**Affiliations:** Lee Kong Chian School of Medicine, Nanyang Technological University, Singapore 308232, Singapore; maurice_vansteensel@ntu.edu.sg

**Keywords:** acne, microbiome, biofilm

## Abstract

This review discusses the microbiology of acne vulgaris, a chronic inflammatory condition of the pilosebaceous unit that affects most adolescents and can persist into adulthood. The current standard of care consists largely of antibacterial interventions, based on the traditional view of *Cutibacterium acnes* as a pathogen. Alternative treatments are suggested by the “comedo switch” hypothesis, which attributes acne to aberrant differentiation of LRIG1+ sebaceous progenitor cells. While there is strong evidence to support this idea, it does not explain the efficacy of antibacterial interventions. We propose a unified mechanism wherein *C. acnes* phylotype IA1 can act as a trigger for the comedo switch. Unlike commensal strains, phylotype IA1 has high lipase activity, hydrolyzing sebum triglycerides into free fatty acids, specifically palmitic acid. This metabolite stimulates LRIG1+ progenitors, inducing inflammation and initial comedo formation. The review discusses *C. acnes* phylotypes, emphasizing known virulence factors of IA1, such as enhanced biofilm formation. We evaluate the efficacy and limitations of both old and new antibacterials, noting how newer materials that selectively remove *C. acnes* IA1 can reduce non-inflammatory acne lesions, supporting a key role for this phylotype in the pathogenesis of acne.

## 1. Introduction

This review discusses the latest insights in the microbiology of acne vulgaris (acne), and what those mean for the treatment of this common skin disease. Acne is a chronic inflammatory condition of the pilosebaceous unit, affecting up to 85% of adolescents and sometimes persisting well into adulthood [1]. While most people have mild disease that eventually resolves, a significant number experience significant inflammation and scarring, in addition to profound psychosocial distress. Medical intervention is often required and will frequently consist of an antibiotic combined with another compound [1]. Considering the rising tide of antimicrobial resistance, as well as the deleterious effects of antibiotic use on the skin and gut microbiome [2], there is a clear need for better and more targeted interventions. To develop those, a detailed understanding of acne pathogenesis is required. This understanding has recently been subject to significant change, and there are presently two viewpoints that so far have been difficult to reconcile. We propose a synthesis, with important consequences for the way we approach acne.

On the one hand, it has long been thought that acne is caused by a commensal turned pathogen, *Cutibacterium acnes*, previously known as *Propionibacterium acnes* [3]. The idea was that excess sebum production at the onset of puberty favors the growth of *C. acnes*, which would then induce follicular hyperkeratinization and, subsequently, comedones (blackheads) through the production of pro-inflammatory metabolites (for instance, [4]). This proposed sequence of events, whilst superficially plausible, leaves crucial observations unexplained. For instance, the almost invariable atrophy of sebaceous glands associated with comedones [5]. The ubiquitous presence of *C. acnes* on both healthy and acne-affected skin is also difficult to reconcile with a straightforward relation to the disease [3].

On the other hand, there is the more recent comedo switch hypothesis, which has gained traction as it is supported by an extensive body of human genetics and cell biology data (reviewed in [6]). In this scenario, Lrig1+ sebaceous progenitor cells in the junctional zone (where the sebaceous duct meets the hair follicle) fail to differentiate and migrate to their eventual fate in the sebaceous gland. Instead, they become “stuck”, adopt an infundibular fate and keep dividing to form a comedo [7]. Because they no longer contribute to the sebaceous gland, the latter shrinks because it is no longer replenished, holocrine secretion progressively reducing the number of cells. Sebaceous gland atrophy leads to abnormal sebum composition, promoting the growth of pro-inflammatory *C. acnes* phylotype IA1, which then causes inflammation [8].

While the comedo switch hypothesis is underpinned by convincing data and has strong explanatory power, it does not explain what triggers the aberrant signaling that causes the switch. It does not support a causal role for *C. acnes,* or for inflammation, which contradicts the known efficacy of antimicrobials. In this review, we will make a case that *C.* *acnes* might have a role in the comedo switch, making it (once again) a target for interventions. The argument will be supported with a comprehensive, but compact overview of the recent literature and other information on *C. acnes’* role in acne and acne pathogenesis.

## 2. *Cutibacterium acnes*

The recent reclassification of *Propionibacterium acnes* to another genus, *Cutibacterium* [9], has helped to better understand the connection between *C. acnes* and skin health. Sequencing and phylogenetic studies have revealed that *C. acnes* is more closely related to other skin-associated bacteria than to other members of the *Propionibacterium* genus. Those are typically not found in the skin, but in other environments such as dairy products. In contrast, *C. acnes* is primarily found on human skin [9].

### 2.1. Phylotypes (Table 1)

Before genetic sequencing became widely available in the 1970s, different strains of *C. acnes* (then called *P. acnes*) were distinguished by serotyping. This technique revealed the existence of at least two distinct groups, called type I and type II [10]. Type I strains were more often found in acne lesions, type II in healthy skin. Of interest, Webster and Cummins reported in 1978 that *C. acnes* type I is more sensitive to lysis by bacteriophages [11].

After PCR technology was introduced and sequencing became more accessible, analysis of the bacterial *recA* and *tly* genes revealed the existence of a third subtype, which was (obviously) named type III. The sequences also suggested that type I could be further split into two subtypes (IA and IB, [12]). The analysis of these single loci was in 2010 superseded by Multilocus Sequence Typing (MLST), where seven–nine housekeeping genes were analyzed simultaneously [13]. MLST resolved type I into phylotypes IA1, IA2 and IB. Further distinctions have been made into clonal complexes and ribotypes [14], but those are not commonly used in clinical studies as their relevance to skin health remains to be determined. 

**Table 1:** Historic overview of *C. acnes* phylotype discovery.

**Phylotype**	Primary discovery method	Key characteristics
**Type I**	Serotyping (Johnson & Cummins, 1972 [10])	Predominant on face/scalp; associated with acne vulgaris.
**Type II**	Serotyping (Johnson & Cummins, 1972 [10])	Often found on trunk; associated with healthy skin or deep-tissue infection (implants).
**Type III**	*recA* sequencing (McDowell, 2005 [12])	Rare; often found on the trunk; distinct elongated shape; may be associated with progressive macular hypomelanosis.
**Subtypes IA/IB**	Multilocus sequence typing (Lomholt, 2010 [13])	Differentiates virulent acne strains (IA) from commensal strains (IB).

The discovery of different phylotypes with distinct characteristics within one species led to a more nuanced understanding of *C. acnes*’ role in acne inflammation. Laboratory data have consistently identified phylotype IA1 as the predominant strain associated with acne inflammation [15]. In one study it made up more than 70% of the total population, with minor contributions from phylotypes IA2, IB, IC and II [16]. Phylotype II is typically associated with healthy skin, but has been known to cause deep-tissue infections, such as those associated with orthopedic implants [17]. The association of phylotype II with healthy skin suggests a commensal role, possibly in competition with the pro-inflammatory phylotype IA1 bacteria. Finally, phylotype III is mostly found on the trunk and does not have a clear association with either acne or skin health. Instead, it has been implicated in the pigmentary condition progressive macular hypomelanosis (PMH) [18].

The “cutitype” concept has been proposed to describe individuals based on their dominant *C. acnes* population, with distinct cutitypes correlating with specific skin states. The loss of phylotype diversity, rather than the mere presence of the bacterium, is a hallmark of acneic skin. In severe inflammatory acne, the diversity of the *C. acnes* population collapses, leading to a dominance of virulent IA1 [16]. This concept aligns with the recent identification of global “dermotypes”, distinct microbiome compositions that vary across different body sites and are characterized by the presence of one or more core species [19]. Of relevance to the present discussion, two forehead dermotypes were identified, distinguished by a relative abundance of *C. acnes* and *Staphylococcus epidermidis*. The study in question did not determine *C. acnes* phylotypes, and it is conceivable that adding this level of classification would have identified additional forehead dermotypes. The interaction between microbiome and host raises the tantalizing possibility of modulating dermotypes for health benefit. We will later see that this seems to be a valid concept.

### 2.2. Virulence Factors and Mechanisms

*C. acnes* phylotype IA1 seems to be the major driver in acne inflammation [8]. This phylotype is thought to be pro-inflammatory because of some key differences in metabolism and behavior compared with the other types. *C. acnes* phylotype IA1 may interact with other bacterial species in the microbiome in a different way from the other phylotypes. This may result in additional pathogenic effects, for example by depriving the host of beneficial metabolites made by suppressed commensals, or by promoting the growth of pathogenic bacteria. The following are known or thought to be involved in type IA1 pathogenicity (Table 2).

#### 2.2.1. CAMP and Hyaluronidase

The literature on these two supposed virulence factors is contradictory. The papers that originally identified CAMP genes, respectively hyaluronidase variants in *C. acnes*, seem to contradict later work, which has generally ascribed considerable importance to them. Valanne et al. in 2005 identified 5 homologs of the Streptococcus agalactiae co-haemolytic Christie-Atkins-Munch-Peterson (CAMP) factor [20]. This secreted protein forms transmembrane pores and contributes to the haemolytic behavior of Streptococcus agalactiae. CAMP factor genes were identified in type IA, IB and II *C. acnes* and, by analysis on blood agar, were found to be active in all three. Type IB and II strains produced mostly CAMP1, whereas type II isolates were found to secrete mostly CAMP2. Interestingly, according to this study type IA *C. acnes* did not produce CAMP in any significant amount. This observation seems to directly contradict the much later literature, which attributes pathogenicity to CAMP expression, albeit without ever demonstrating directly any plausible mechanism in an accurate model. For instance, Chen et al. found that some CAMPs could induce IL6 and IL8 expression in mouse skin and interpreted the resulting inflammation as indicative of CAMPs contributing to acne [21].

The situation for hyaluronidase is similarly confusing. The paper originally identifying the two variants provides evidence that the *C. acnes* phylotype IA expresses a variant with lower activity [22], directly contradicting a later paper that states the exact opposite [23]. If one stops to consider, there seems to be little utility in expressing this enzyme for type I *C. acnes*, which resides exclusively on the skin surface. Type II, which can cause deep-tissue infections, has much more use for it.

#### 2.2.2. Lipase Activity and Sebum Hydrolysis

The *C. acnes* lipase gene gehA (glycerol-ester hydrolase A) is conserved across all phylotypes, but its expression and the enzymatic efficiency vary between strains. Compared to phylotype II strains, phylotype IA1 have higher lipase activity in culture [24]. This enzyme hydrolyzes sebum triglycerides into free fatty acids (FFAs) and glycerol. Glycerol can serve as a carbon source for the bacterium, while the FFAs contribute to the acidification of the skin surface. A lower skin pH inhibits the growth of pathogenic competitors like *Staphylococcus aureus*. In the context of acne, specific FFAs, particularly palmitic acid (C16:0), have been hypothesized to act as pro-inflammatory signals and possible modulators of keratinocyte differentiation [25]. As we will see, recently published work lends credence to this hypothesis.

#### 2.2.3. Porphyrin Production and Oxidative Stress

Bacteria use heme to obtain iron, after which it is degraded. Porphyrins are intermediate products in that degradation, as they are in humans. *C. acnes* phylotype IA1 strains produce significantly higher levels of porphyrins compared to type II strains, particularly coproporphyrin III [26]. Porphyrins can catalyze the production of reactive oxygen species (ROS) when exposed to environmental stressors or UV light [27]. ROS induce oxidative stress in follicular keratinocytes, triggering the release of pro-inflammatory cytokines such as IL-8 [28]. ROS can also convert squalene in sebum to comedogenic squalene peroxides. As an aside, this is why some porphyrias, inborn errors of heme metabolism, can cause severe skin symptoms including inflammation and scarring. It also provides an explanation for the known worsening of acne by sun exposure [29]. Of interest, the production of porphyrins may be modulated by host factors. Vitamin B12 supplementation has been shown to alter the transcriptional profile of *C. acnes* in healthy subjects, repressing the synthesis of vitamin B12 (which the bacterium can produce endogenously) and potentially redirecting metabolic flux toward porphyrin biosynthesis [30]. One of the ten subjects in this study developed acne within a week of starting the supplementation. This observation is consistent with previously published work defining “cobalamin-associated acne,” where B12 supplements trigger flare-ups in susceptible individuals [31].

#### 2.2.4. Adhesion and Biofilm Formation

The formation of biofilms is thought to be a key factor in making acne a chronic disease that can take a long time to respond to treatment—clinical experience tells us that antibiotics need to be administered for at least three months, sometimes longer [32]. Biofilms are complex aggregates of bacteria (either a single species, or several) embedded in a self-produced extracellular polymeric substance (EPS), primarily composed of glycosaminoglycans, proteins, and extracellular DNA [33].

In vitro assays demonstrate that phylotype IA1 strains isolated from acne lesions are significantly more likely to produce biofilms than phylotype II strains [34]. This enhanced biofilm capacity may enable IA1 strains to adhere to the follicular wall and protects them from antimicrobials (including those made by other bacteria) and immunological surveillance. The biofilm has also been hypothesized to function as a biological adhesive; by binding corneocytes (keratinocytes) together, the biofilm may prevent or delay normal desquamation, possibly contributing to comedo (blackhead) formation [35].

However, there is no strong evidence for the in vivo pathogenicity of *C. acnes* biofilms. There is only one paper, published in 2014, that provides some data in support [36]. In transversely sectioned skin biopsies, biofilms were seen to adopt different distributions, where they were either first observed in the top layer of the stratum corneum, or appeared from 80 μm below the surface. Biofilms were attached to the follicle wall, or else to the hair shaft. In this study, sebaceous glands were always free of bacteria, consistent with earlier work [37] but in contrast to what has been claimed in some highly cited reviews (for instance, [38]). It is important to note that the skin samples were taken from excisions of melanocytic nevi, on the face, back and abdomen of individuals who did not have acne. Also, biofilms were only observed in samples from two out of six individuals. The work did not characterize the phylotype of the bacteria in the biofilm.

#### 2.2.5. Extracellular Vesicles (EVs)

Not too long ago, it was discovered that *C. acnes* releases extracellular vesicles (EV), also known as outer membrane vesicles or membrane vesicles [39]. These are lipid bilayer structures between 30 and 1000 nm in diameter that carry proteins, nucleic acids, metabolites and other bioactive molecules. EVs are produced by prokaryotes, archaea and eukaryotes, suggesting that they represent a staggeringly ancient mechanism of great importance.

Choi et al. reported that EV isolated from *C. acnes* could induce an “acne-like” phenotype in normal human epidermal keratinocytes (NHEK) and reconstituted human skin, consisting of keratinocyte hyperproliferation and changes in the expression of some epidermal markers like KRT10 and DSC1. While that hardly seems to qualify as “acne-like”, the EVs did induce the expression by isolated NHEK of pro-inflammatory cytokines known to promote neutrophil migration in a TLR2-dependent mechanism. Thus, a connection between *C. acnes* EV secretion and acne inflammation seems possible.

In 2022, Chudzik et al. compared EVs derived from *C. acnes* phylotype IA, IB and II, assessing morphology as well as lipid and protein content [40]. By SDS-PAGE, there were clear differences in the protein content between EVs isolated from each strain. Type IA1 produced vesicles with an abundance of proteins between 15 and 75 Kda, whereas type IB seemed to contain only a few proteins. Type II was intermediate. Lipid profiles likewise were different, with type IB having the most lipid compounds. The paper did not characterize any of the proteins or lipid, so one can only conclude that the different phylotypes produce different EVs, which may well be related to their different behavior and virulence in human skin.

Cheung et al. demonstrated that EVs produced in vitro by *C. acnes* phylotype 1A1 from inflammatory acne lesions significantly increased the production by keratinocytes of pro-inflammatory cytokines and anti-microbial peptides, compared with EVs derived from phylotype 1A1 isolated from normal human skin [41]. This finding implies that the inflammatory microenvironment of the acne lesion may modify the virulence of the bacterium, causing it to secrete more potent inflammatory mediators into its EVs. This would then create a feed-forward loop between the host and the bacterium that may well contribute to acne’s chronic course.

**Table 2:** Comparative virulence profiles of *C. acnes* phylotypes.

Feature	Phylotype IA1 (Acne-Associated)	Phylotype II (Health-Associated)	Clinical Implications
Primary niche	Sebaceous follicle (Lipid-rich)	Skin surface/deep tissue	IA1 dominates the acne lesion environment.
Sebum metabolism	High lipase activity; robust growth in sebum mimics	Moderate lipase activity; slower growth	IA1 generates higher levels of pro-inflammatory FFAs.
Porphyrin levels	High; responsive to B12	Low; less responsive to B12	High ROS generation drives keratinocyte stress and inflammation
Biofilm capacity	Strong/dense producer	Weak/moderate producer	Biofilm suggested to act as “glue” for comedone formation; may reduce efficacy of antibiotics, may interfere with healthy microbiome; may contain inflammatory mediators
Host Adhesion	High affinity for sebocytes/keratinocytes	Low affinity	Direct contact facilitates invasion and signaling; little evidence for in vivo relevance

## 3. *Cutibacterium acnes* and Comedogenesis

As outlined above, most published data point to a causal role for *C. acnes* phylotype IA1 in acne inflammation. However, inflammation is not how the condition is thought to start. The initial, hallmark lesion of acne is the comedo (blackhead). There is strong and growing evidence for understanding this lesion as a cystic expansion of the junctional zone, caused by abnormal differentiation and proliferation of the sebaceous progenitor cells that live there. Both human genetics and cell biology overwhelmingly point to a role for Wingless signaling and tissue remodeling in producing a comedo [5,6,7,42]. There seems to be little space for *C. acnes*, which was for a long time thought to be a major causal factor in comedogenesis. Most loci indicated by GWAS do not have an obvious relation with immunity or inflammation [6]. Intriguingly, recently published data now see the pendulum swing back. The new insights point to a mechanism by which *C. acnes* would, after all, be able to at least indirectly trigger a comedo switch.

### Lrig1+ Progenitor Cells, the Comedo Switch and Fatty Acids

The pilosebaceous unit is maintained by distinct populations of stem and progenitor cells. Of particular importance for our present discussion is the Lrig1+ (Leucine-rich repeats and immunoglobulin-like domains protein 1) progenitor cell pool, located in the follicular isthmus and the junctional zone, where the sebaceous duct meets the hair follicle. This population is referred to as progenitors, since they are already committed—these cells can assume either an infundibular or a sebaceous fate [43]. They are necessary for growth and homeostasis of the sebaceous gland, because sebum is produced by holocrine secretion, a process that bears more than a passing resemblance to epidermal differentiation. Instead of producing a cornified lipid envelope like keratinocytes do, sebocytes progressively become filled with lipid droplets as they differentiate towards the center of the gland.

Just like the epidermis, sebaceous glands have a basal layer of transit amplifying cells that need to be replaced from a stem or progenitor cell pool. For the sebaceous gland, these progenitors are the Lrig1+ cells in the junctional zone. These cells replenish the gland under homeostatic conditions, and are responsible for its growth at the onset of puberty. Since the progenitors are in the junctional zone, they will have to migrate to the gland whilst undergoing sebaceous differentiation. Others and we have shown that the Lrig1+ progenitors are controlled by Wingless signaling, the intensity of the signal controlling the eventual fate decision [42,44,45]. According to the comedo switch hypothesis, which was first formulated by Saurat in 2015, comedogenesis starts when instead of adopting either a sebocyte or a keratinocyte fate in an orderly fashion, progenitors undergo aberrant proliferation and differentiation [7]. They no longer migrate, which results in expansion of the junctional zone that gets filled with a mix of keratins and lipids. As the sebaceous gland will no longer be replenished, it will become atrophic and indeed, sebaceous glands associated with comedones are always atrophic ([46].

There is abundant support for this scenario from genetics, molecular biology and clinical data [6]. Individuals with severe acne have a larger burden of genetic risk factors, hence are more easily triggered by whatever environmental factor capable of doing so. The question, of course, is what those factors are.

Sugihira et al. provide evidence that palmitic acid (C16:0), a saturated fatty acid, is elevated in sebum from people with acne, with levels correlating with inflammatory lesion count [47]. In mouse experiments, palmitic acid was found to penetrate the epidermis, where it drove neutrophil recruitment by Lrig1+ progenitor cells and, importantly, increased proliferation of these cells with sebaceous differentiation. In genetically susceptible individuals, this differentiation step could go awry and turn into a comedo switch. Intriguingly, a recent GWAS identified *FASN* as an acne risk factor [48]. *FASN* codes for an enzyme expressed in sebocytes that synthesizes palmitic acid. This beautiful convergence of data strongly argues for its real-life relevance. However, Sugihara et al. note in their paper that their data were generated in germ-free mice. At first sight, this might seem to disqualify *C. acnes*. However, as noted above, lipase activity in *C. acnes* phylotype IA1 is higher than in non-acne associated strains, and lipase breaks down triglycerides into glycerol and free fatty acids, of which palmitic acid is one. Thus, a coherent sequence of events emerges, in which acne results from an interplay between host factors and environmental triggers, with a prominent role for *C. acnes* phylotype IA1 in driving the disease:

1. Androgen stimulation: puberty triggers androgen production (no androgens, no acne [49]), stimulating the sebaceous gland to synthesize triglycerides. If the host has a *FASN* risk allele, concentrations of palmitic acid may be high to begin with.
2. Bacterial hydrolysis: *C. acnes* IA1 colonizes the follicle. Its lipases hydrolyze these triglycerides to release free fatty acids, including palmitic acid (C16:0).19
3. Progenitor cell activation: palmitic acid activates Lrig1+ progenitors in the junctional zone.
4. Inflammation and the comedo switch: Lrig1+ cells undergo the comedo switch in genetically susceptible individuals and produce cytokines that attract neutrophils and monocytes. The sebaceous gland associated with the affected hair follicle becomes atrophic for lack of replenishment.
5. Niche expansion: the comedo with its abnormal sebum content creates the ideal environment for *C. acnes* IA1 expansion and biofilm formation, resulting in accelerated palmitic acid production and still stronger stimulation of the progenitor compartment.

With *C. acnes* back in the spotlight as a (co-) causal factor in the pathogenesis of acne, antibacterial treatments make more sense than ever. However, currently available solutions are hardly optimal.

## 4. Antibacterial Interventions

Antiseptics like benzoyl peroxide are typically used for mild to moderate acne. Broad-spectrum antibiotics, either topical or systemic, are reserved for moderate-to-severe cases where there is a significant risk of scarring [32]. Both categories of antibacterials will be briefly discussed here, with a focus on their mechanism of action and the side effects that necessitate a critical re-evaluation of their use.

### 4.1. Antibiotics: Tetracyclines and Macrolides

Systemic and topical antibiotics, particularly tetracyclines (doxycycline, minocycline, sarecycline) and macrolides (erythromycin, azithromycin), have been a mainstay of moderate-to-severe acne treatment for a long time [32]. Tetracyclines are bacteriostatic and work by binding reversibly to the 30S ribosomal subunit in the target bacteria. This blockade prevents the association of aminoacyl-tRNA with the bacterial ribosome, inhibiting protein synthesis [50]. In addition to their antibiotic effects, tetracyclines are known to inhibit certain bacterial and host metalloproteinases (MMPs) [51] and downregulate neutrophil chemotaxis [52]. Macrolides (erythromycin, azithromycin, clarithromycin) bind to the 50S ribosomal subunit at the 23S rRNA component, blocking the polypeptide exit tunnel and halting translation [53]. Based on this mechanism of action, macrolides are bacteriostatic just like tetracyclines. They also have anti-inflammatory properties, being able to reduce expression of several cytokines such as IL4, -5, and -17 [54].

The widespread use of these antibiotics for a common disorder like acne has measurably contributed to the increasingly dire problem of antimicrobial resistance [55]. The effects are already a problem for dermatology-resistance rates for erythromycin in *C. acnes* now exceed 50% in many regions, rendering it largely obsolete as a monotherapy [56]. Apart from contributing to antimicrobial resistance, the systemic administration of broad-spectrum antibiotics induces profound and enduring dysbiosis in the gut as well as in the skin [55]. Even short-term antibiotic use significantly reduces gut microbial diversity for a considerable time, depleting beneficial genera such as Bifidobacterium and Lactobacillus [57]. This dysbiosis has been epidemiologically linked to an increased risk of inflammatory bowel disease (IBD) and metabolic disturbances [57].

Topical antibiotics are not harmless in that respect. They can select for resistant *Staphylococcus epidermidis* strains, which may serve as a reservoir for resistance genes that can be horizontally transferred to more pathogenic *Staphylococci* [58]. Moreover, whilst topical antibiotics do suppress *C. acnes*, they do not discriminate between phylotypes, plus they affect the entire facial skin microbiome. Hence, they do not address, and might even worsen, the dysbiosis that is characteristic of acne. This is probably one reason why both systemic and topical antibiotics tend to act slowly on acne inflammation—another reason might be the relative inaccessibility of *C. acnes* IA1 in its biofilm.

### 4.2. Antiseptics: Benzoyl Peroxide (BPO)

Benzoyl peroxide (BPO) is an organic peroxide. Upon application to the skin, it is absorbed and converted into benzoic acid, ~5% of which enters the systemic circulation and is excreted [59]. Benzoic acid in the skin reacts with cysteines, generating oxygen free radicals which oxidize bacterial proteins and cell membrane lipids, rapidly killing bacteria [60]. Unlike antibiotics, BPO does not rely on a specific target, meaning *C. acnes* cannot develop resistance to it. Hence, BPO is commonly used in combination with antibiotics to prevent resistance, with the antibiotics used for their anti-inflammatory effects [32].

Though effective, BPO has significant drawbacks. First, it kills bacteria indiscriminately. It will come as no surprise that BPO treatment significantly reduces microbial alpha diversity on the skin [61]. Hence, it does not address the dysbiosis in acne any more than antibiotics do. Second, as a source of oxygen free radicals, BPO depletes skin antioxidants (like Vitamin E) and damages the skin’s lipid barrier [62]. This manifests clinically as dryness, erythema, and scaling. Crucially, a compromised barrier could induce a compensatory inflammatory response, counteracting the anti-inflammatory goals of the treatment.

### 4.3. Microbiome-Modulating Interventions

The significant drawbacks of traditional antimicrobials are incentivizing the development of interventions to edit or modify the microbiome. As opposed to the indiscriminate killing by antibiotics or antiseptics, these novel interventions aim to selectively remove pathogenic or otherwise undesirable bacteria, and/or to restore eubiosis. In the case of the latter, it is thought that restoring normal relations in a population can suffice to suppress “bad” bacteria, or mitigate the consequences of their presence. A few examples that are supported by published data will be mentioned here.

### 4.4. Pre- and Postbiotics

Prebiotics are nutrients that are meant to promote the growth or function of a specific bacterial population. In case of *C. acnes*, there is an interaction with *S. epidermidis* whereby the latter ferments glycerol into succinic acid, which inhibits *C. acnes* growth and inflammation [63]. Hence, prebiotic formulations containing succinic acid or substrates that promote *S. epidermidis* growth are being developed to restore the normal balance between this species and *C. acnes*, which is disrupted in acne [63].

Postbiotics are bacterial metabolites or extracts that induce biological activity in the host. In a small study, topical treatment with a ferment lysate of *Lactobacillus plantarum* (VHProbi E15) demonstrated a significant reduction compared to baseline in acne lesion count, sebum production, and erythema after 4 weeks [64]. The mechanism of action is not clear. It might be speculated that the lysates contain bacterial cell wall components that stimulate Toll-like Receptors (specifically TLR2) to modulate the immune response away from the Th17 pathway typically activated by *C. acnes*. Alternatively, chronic TLR2 activation might influence the interaction between the skin and its microbiome, for instance by causing the skin to produce antimicrobial products [65].

Whilst theoretically (and commercially) attractive, both prebiotics and postbiotics have drawbacks that may well prevent them from becoming mainstream treatments for acne. In the case of prebiotics, the interactions between the species in a microbiome are insufficiently understood to predict the effects of stimulating the growth one or more of them. Similarly, the evidence in favor of probiotic efficacy is still weak [66]. A similar problem hampers the use of postbiotics, where the effects of various bacterial metabolites on the skin or its microbiome are not known, and where the composition of any ferments is often not fully known either. In addition, there are significant batch effects, in particular with ferments [66].

### 4.5. Bacteriophage Therapy

Neither pre- nor postbiotics are in any way targeted interventions, and their ultimate effects remain unpredictable. For that reason, and to avoid the issue of antimicrobial resistance, scientists and clinicians are increasingly looking to bacteriophage and their endolysins [67].

Bacteriophages (phages) are viruses that prey on bacteria. In the so-called lytic part of their life cycle, phages kill their host using peptidoglycan hydrolases known as endolysins. Phages can in principle target a single species of bacteria, thus leaving the rest of the microbiome intact [68]. Importantly, they are able to penetrate bacterial biofilms and do not cause resistance as quickly as antibiotics [69]. As mentioned, *C. acnes* type I and II phylotypes differ in their sensitivity to phage infection, offering the possibility of selective strain removal [11].

In view of these advantages, it is of interest to examine the available data from the phase 2 trial of BX001, a topical gel containing a cocktail of naturally occurring *C. acnes* phages, conducted by the biotech company BiomX [70]. The trial was a randomized, double-blind, vehicle-controlled study involving 140 subjects with mild-to-moderate acne vulgaris. In in vitro studies, the phage cocktail reduced *C. acnes* levels significantly compared to baseline. Clinically, the treatment arm showed a strongly reduced the number inflammatory lesions, with an improved the Investigator’s Global Assessment (IGA) score. However, the control arm showed similar efficacy, (https://www.biospace.com/biomx-reports-topline-results-of-phase-2-cosmetic-acne-study, accessed on 11 February 2026) possibly because the gel vehicle provided moisturization, or (more likely) contained excipients with antimicrobial properties.

### 4.6. Endolysins

Bacteria can develop resistance to bacteriophages, and the consistent, GMP-compliant production of phage (cocktails) remains a significant challenge [71]. There are also regulatory hurdles involved in the use of replicating viruses that are able to integrate into bacterial genomes and thereby potentially transfer genetic material [72]. Therefore, there is strong interest in using the endolysins that are produced by the phage at the end of its lytic cycle to burst the host cell [73]. It turns out that recombinant endolysins can be applied topically to lyse bacteria from the outside in, even though they are meant to work from the inside out [74]. Because endolysins kill by disrupting the bacterial cell wall and do so rapidly, bacteria are unlikely to develop resistance. They are also safe, as they do not interact with any other structure than the bacterial cell wall. Finally, being water soluble but also compatible with various anhydrous vehicles, endolysins are easy to formulate.

Given these advantages, there have been several attempts to develop (recombinant) endolysins targeting *C. acnes* for either clinical or personal care use. However, to date there have been no reports of successful clinical use. Based on the available literature as well as our own experience, this likely reflects the relatively low lytic activity and specificity of *C. acnes* endolysins [75]. It should also be noted here that endolysins cannot distinguish between phylotypes, as the relevant genetic differences do not affect cell wall structure.

In view of endolysin’s lack of efficacy, it is worth noting here that the Singaporean biotech startup ArrowBiome has developed a material (SmartArrow^TM^) based on the cell wall binding domain of a lysin produced by a *C. acnes* bacteriophage. The company claims that it physically disrupts biofilms formed by *C. acnes* phylotype IA1 and shows strong efficacy in reducing inflammatory as well as non-inflammatory acne lesions (www.arrowbiome.com).

## 5. Conclusions

In conclusion, the latest data on the microbiology of acne support a key role for *C. acnes* phylotype IA1 in the pathogenesis of acne. I propose that a key factor is its production of (C:16) palmitic acid, and it will be of considerable interest to test this hypothesis in future trials. This notion is compatible with Cheung et al.’s finding that *C. acnes* type IA1 isolated from people with acne is a significantly stronger inducer of inflammation than the same phylotype isolated from healthy skin [41]. The extent to which *C. acnes* type IA1 forms biofilms in vivo, and the extent to which those contribute to its pathogenicity remain to be fully elucidated. Certainly the results claimed by ArrowBiome suggest that biofilm formation is a crucial factor in acne inflammation, and should be studied in more detail both in vitro and in vivo.

The genetics of acne unequivocally indicate the importance of host factors in inducing a comedo switch. As noted, the microbiome factors are presently not entirely clear, and larger datasets are needed to address whether there are people with acne who have predominantly *C. acnes* type II on their skin rather than type I. Presumably, they would need a different therapeutic approach focused more on host factors such as lipid production, for instance with a FASN inhibitor such as Denifanstat [76]. Interestingly, whilst this drug has proven to be effective in reducing total and inflammatory lesion counts, its overall efficacy in these parameters at three months is less than that of benzoyl peroxide. This observation might be taken to support the notion that patient selection based on microbiome composition is necessary. Alternatively, it might mean that endogenous palmitic acid production does not make a major contribution to the total.

Finally, this review has not touched upon the many denizens of the skin microbiome that are not bacteria: eukaryotes such as fungi, archaea and viruses. Their role in acne, if any, remains to be addressed, which will require expensive shotgun-metagenomics. The cost may well be justified by the benefits that could be reaped from a more complete understanding of microbiome complexity in acne versus healthy skin. Knowing which components of the microbiome are essential in this diversity will be of considerable help when designing future microbiome-friendly interventions.

## Data Availability

Not applicable.
